# Small cell lung cancer growth is inhibited by miR-342 through its effect of the target gene IA-2

**DOI:** 10.1186/s12967-016-1036-0

**Published:** 2016-09-26

**Authors:** Huanyu Xu, Tao Cai, Gilberto N. Carmona, Liron Abuhatzira, Abner L. Notkins

**Affiliations:** Experimental Medicine Section, Laboratory of Sensory Biology, National Institute of Dental and Craniofacial Research (NIDCR), National Institutes of Health (NIH), B30/Rm106, Bethesda, MD 20892 USA

**Keywords:** Small cell lung cancers (SCLC), Dense-core vesicle (DCV), microRNA, Autocrine, Therapeutic approach

## Abstract

**Background:**

Small cell lung cancers (SCLC) are tumors of neuroendocrine origin. Previous in vitro studies from our laboratory showed that SCLC expresses high levels of the transmembrane dense core vesicle protein IA-2 (islet cell antigen-2) as compared to normal lung cells. IA-2, through its effect on dense core vesicles (DCVs), is known to be involved in the secretion of hormones and neurotransmitters. It is believed that the dysregulated release of the neurotransmitter Acetylcholine (ACh) by DCVs has an autocrine effect on SCLC cell growth. Recently, we found that IA-2 is a target of the microRNA miR-342 and that miR-342 mimics suppress the expression of IA-2. The present experiments were initiated to see whether IA-2 and/or miR-342 affect the growth of SCLC.

**Methods:**

SCLC cell growth was evaluated following the knockdown of endogenous IA-2 with RNAi or by overexpressing miR-342 with a mimic. The secretion and content of ACh in SCLC cells was analyzed using a human acetylcholine ELISA (enzyme-linked immunosorbent assay) kit.

**Results:**

The knockdown of endogenous IA-2 by RNAi reduced SCLC cell growth within 4 days by 40 % or more. Similar results were obtained when these cell lines were transfected with a miR-342 mimic. The knockdown of IA-2 by RNAi or miR-342 with a mimic also resulted in a significant decrease in the secretion of ACh, one of the autocrine hormones secreted by SCLC. Further studies revealed that the growth of SCLC cell lines that had been treated with the miR-342 mimic was restored to nearly normal levels by treatment with ACh.

**Conclusion:**

Our studies show for the first time that both miR-342 and its target gene IA-2 are involved in the growth process of SCLC cells and act by their effect on autocrine secretion. These findings point to possible new therapeutic approaches for the treatment of autocrine-induced tumor proliferation.

## Background

Lung cancer is divided into two histopathological types: non-small cell lung cancer (NSCLC) accounting for ∼80 to 85 % of lung cancers, and small cell lung cancer (SCLC) which accounts for ∼15 to 20 % of lung cancers [1–4]. SCLC has distinctive neuroendocrine features [5] and is capable of secreting or co-secreting a variety of neuropeptides and neurotransmitters including acetylcholine (ACh) [6, 7], pro-opiomelanocortin (POMC), and adrenocorticotropic hormone (ACTH) [8, 9]. Of the various neuropeptides, ACh, which is an autocrine growth factor, facilitates SCLC growth [6, 7].

IA-2 (also known as islet cell antigen 512 [ICA512] or protein tyrosine phosphatase, receptor type N [PTPRN]) is an integral transmembrane protein of dense core vesicles (DCV) [10, 11], and plays an important role in the secretion of hormones and neurotransmitters such as insulin, luteinizing hormone (LH), follicular stimulating hormone (FSH), norepinephrine (NE), dopamine, and renin [10, 12–17]. In addition, IA-2 is highly expressed in tumors of neuroendocrine origin [18, 19], including SCLC tumors, as observed by northern blot analysis [18, 19]. IA-2, together with its paralog protein IA-2β (also known as phosphatase homologue in granules of insulinoma [Phogrin] or protein tyrosine phosphatase, receptor type N2 [PTPRN2]), has been implicated in the growth of pancreatic β cells [20, 21]. Collectively, these studies suggest that IA-2 may be associated with the pathogenesis of SCLC, specifically in respect to the regulation of the neuroendocrine secretion.

The role of microRNAs (miRNAs) in tumor growth is being widely studied [22]. It is believed that the dysregulation of some miRNAs is linked to lung cancer, and that quantification of miRNA expression can be used to study disease prognosis. In this context, a recent microarray analysis showed that miR-342 is one of the most downregulated miRNAs associated with lung cancer [23]. The underlying role of miR-342 in the pathogenesis of lung cancer, however, remains unclear.

In the present study, we measured the levels of IA-2 and miR-342 in two SCLC cell lines, and then inhibited IA-2 by siRNA transfection and altered miR-342 levels with mimics and inhibitors to determine their effect on SCLC growth and ACh levels.

## Methods

### Reagents and cell lines

Predesigned siRNAs (Hs_PTPRN_3, 4, 5, 6) against human IA-2 (NM_001199763, NM_001199764, NM_002846), scrambled negative control, miR-342 mimic, miR-342 inhibitor, and HiPerFect Transfection Reagent were obtained from Qiagen. For RNA analysis: miRNeasy Mini kit, miScript II RT kit, and miScript SYBR Green PCR kit also were purchased from Qiagen. SYBR Green PCR Master Mix was obtained from Applied Biosystems. The human Acetylcholine (ACh) ELISA Kit was used for determination of endogenous ACh levels (MyBioSource, San Diego, CA). The Cell Counting Kit-8, neostigmine, and Acetylcholine chloride were purchased from Sigma-Aldrich, and the M-PER Mammalian Protein Extraction Reagent was obtained from Thermo Fisher Scientific.

Antibodies for western blotting and immunostaining were purchased from Invitrogen. VECTASHIELD Mounting Medium with DAPI (4′,6-Diamidino-2-Phenylindole, Dihydrochloride) was obtained from Vector Laboratories. Rabbit polyclonal antibody against amino acid residues 65–80 of the *N*-terminal region of IA-2 (GenBank Acc. No., NP_001186692), was used for both Western blotting (1:500) and immunostaining (1:100). Both items were purchased from Abgent, San Diego, CA, USA [24].

SCLC cell lines (NCI-H82 and NCI-H345) were purchased from ATCC. The NCI-H82 cell line was cultured in Dulbecco’s Modified Eagle Medium (DMEM) supplemented with 10 % fetal bovine serum (FBS). The NCI-H345 cell line was cultured in DMEM/Ham’s F-12 (1:1), supplemented with 10 % FBS, insulin (0.005 mg/ml), transferrin (0.01 mg/ml), sodium selenite (30 nM), hydrocortisone (10 nM), beta-estradiol (10 nM), HEPES (10 mM), and l-glutamine (2 mM). Both cell lines were cultured at 37 °C in 5 % CO_2_.

### Immunofluorescence

Transfected NCI-H82 cells (on chamber slides) were fixed in 4 % paraformaldehyde. Primary and secondary antibodies (IA-2; 1:100 dilution), and Alexa Fluor 568 Goat Anti-Rabbit IgG (H+L) (1:500 dilution), respectively, were used. VECTASHIELD mounting medium with DAPI (Vector Laboratories) was used for nuclear staining. Images were captured with ZEN Imaging Software (ZEISS) using constant exposure parameters for each fluorescence channel. Images shown are representative field of three independent experiments.

### IA-2 siRNA transfection

A total of 2 × 10^4^ cells/well were seeded in 24-well culture plates 1 day prior to undergoing transfection with either the IA-2 siRNAs (Hs_PTPRN_3, 4, 5, 6), or the scramble siRNA with the HiPerFect Transfection Reagent for 10 min (according the manufacturer’s instruction). Briefly, cells were resuspended in 100 µl of fresh culture medium (containing serum and antibiotics), and then 100 μl of the siRNA transfection complexes solution (100 nM at final concentration after adding 400 µl culture medium) was added drop-wise onto the cells. The plates were then gently swirled to ensure uniform distribution of the transfection complexes. The suspension was incubated at normal growth conditions for 6 h, and then 400 µl of the culture medium (containing serum and antibiotics) was added to the cells and incubated until further analysis.

**Fig. 1:**
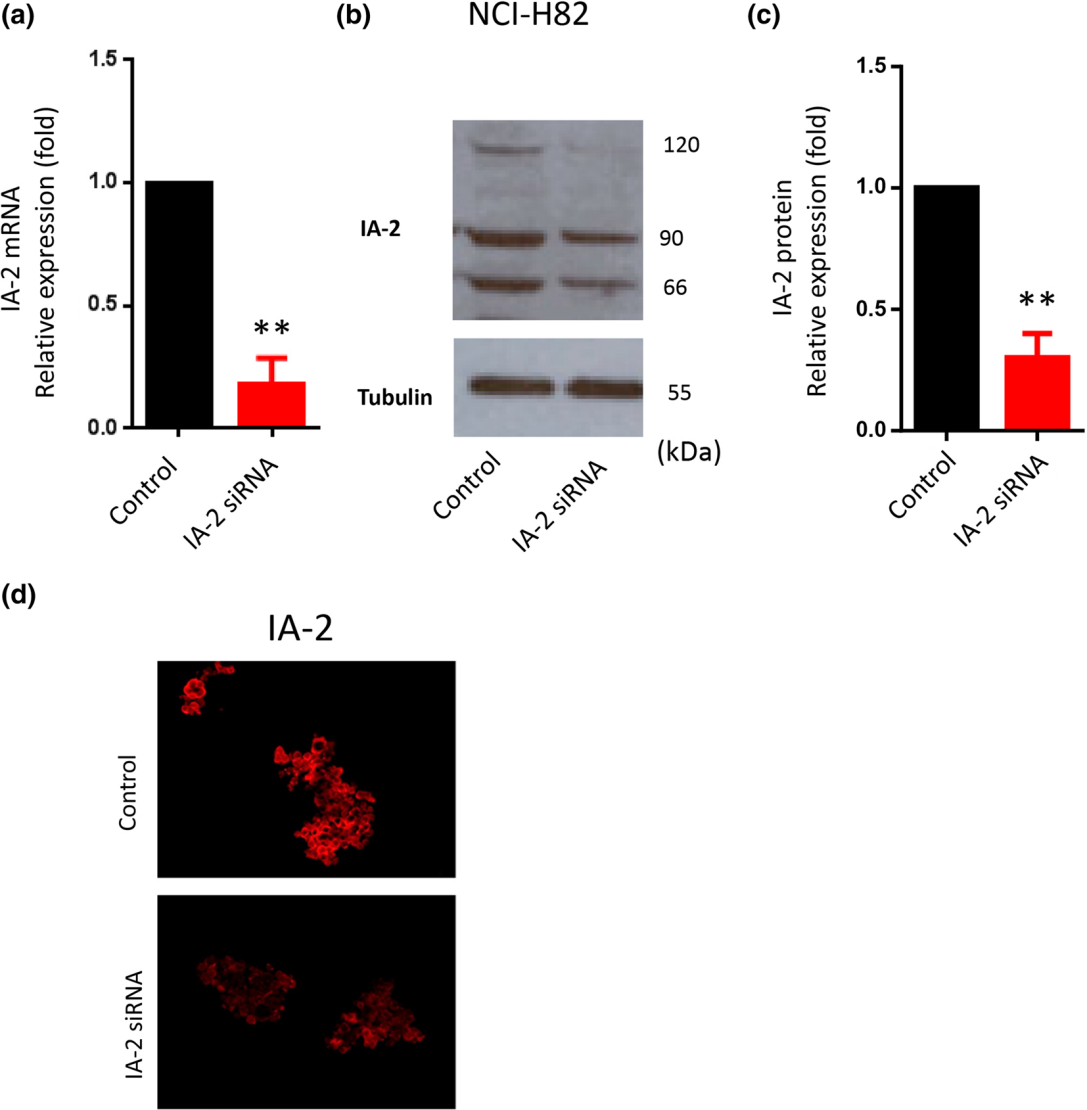
IA-2 expression is decreased by siRNA. **a** The knockdown of endogenous IA-2 mRNA by siRNA (determined by quantitative RT-PCR analysis), was reduced ~80 % in NCI-H82. **b** *Western blot* shows that protein levels for IA-2 (as evaluated by anti-IA-2 N-terminal #49 antibody), was also significantly reduced. Three major bands of IA-2 are detected as indicated due to post-translational modifications [33]. **c** IA-2 protein level was reduced ~70 % as determined by the intensity of the 90-kDa bands using the NIH Image J program. **d** Immunostaining with the IA-2 N-terminal antibody shows that the level of IA-2 in IA-2-specific RNAi treated NCI-H82 cells was significantly lower than the control cells. Data (mean ± SE) were derived from three independent experiments (in triplicate), and normalized to GAPDH mRNA levels. **P < 0.01

### MicroRNA mimic and inhibitor transfection

Cells were transfected with the miR-342 mimic or inhibitor, or with the scramble negative control using the HiPerFect Transfection Reagent (according to the manufacturer’s instructions), and incubated for 10 min further analysis. First, cells (2 × 10^4^ cells/well) were transferred into 24-well plates after overnight culture. The miRNA mimic, miRNA inhibitor, or the control was diluted in 100 μl culture medium without serum (a final miRNA mimic concentration of 5 nM, or a final miRNA inhibitor concentration of 50 nM). Subsequently, the HiPerFect Transfection Reagent (3 μl) was added into the diluted miRNA mimic/inhibitor solution, and then vortexed. After 8 min, the solution was incubated for 10 min at room temperature, and then the complexes were added onto the cells. Finally, the cells were incubated with the transfection complexes under their normal growth conditions until further analysis.

### Cell growth assay

Cell proliferation was tested using the Cell Counting Kit-8 (CCK-8) assay according to the manufacturer’s instructions (Sigma-Aldrich, St. Louis, MO). In brief, after the cells were subjected to the different treatments (transfection with IA-2 siRNA or miR-342 mimic or inhibitor), they were cultured for up to 4 days. Cells were collected each day and then transferred into 96-well plates. Subsequently, CCK-8 (10 µl) was added to each well, and then the cells were cultured for another 3 h. Cell density was determined by measuring the absorbance at 450 nm using a VersaMax Microplate Reader (Molecular Devices, USA).

### ACh secretion and cell content assay

After transfecting the NCI-H82 and NCI-H345 cells with either the IA-2 siRNA, miR-342 mimic, or the miR-342 inhibitor, the cells were incubated for 4 days, and then the cells and supernatants were collected [6, 7]. ACh level was measured using a human acetylcholine ELISA kit according to manufacturer’s protocol. For determination of ACh release, cell suspensions were collected with 5 × 10^−5^ M neostigmine. For measurement of ACh contents, cells were washed in 1× PBS, counted, and then sonicated in ice-cold phosphate buffer (pH 7.4) containing 10^−2^ M of the acetylcholinesterase inhibitor neostigmine. All samples were centrifuged at 1000 rpm for 2 min at 4 °C; supernatants were then immediately assayed for determination of ACh levels.

### Total RNA, miRNA, and quantitative PCR

Total RNA and miRNA were extracted using the miRNeasy Mini kit following the manufacturer’s protocol. Quantitative RT-PCR for both mRNA and miRNA was performed using the miScript II RT kit, and then the miScript SYBR Green PCR kit for measuring miR-342 expression, or SYBR Green PCR Master Mix for measuring the expression of IA-2 and GAPDH (glyceraldehyde-3-phosphate dehydrogenase) mRNA with a 7500 Real-Time PCR system (Applied Biosystems). IA-2 primer sequences used in PCR analysis, forward: 5′-ACGCTCACGCAGTTCCACTT-3′, reverse: 5′-GCGATGTCAATCTCCTTCAC-3′; GAPDH primer sequences, forward: 5′-GAGAACGGGAAGCTTGTCAT-3′ reverse: 5′-CAGAGATGATGACCCTTTTGG-3′.

### Protein extraction and western blot analysis

Proteins from cells were isolated by M-PER Mammalian Protein Extraction Reagent. Protein concentrations were quantified; samples were mixed with reducing loading buffer, and then heated at 70 °C for 10 min. Equal amounts of protein was loaded into each lane, and separated by electrophoresis. Proteins were subsequently transferred onto a PVDF (polyvinylidene fluoride) membrane and then blocked in blocking buffer (PBS/0.1 % Tween20 (PBS-T)/5 % nonfat-milk). After blocking for 2 h, blots were incubated over night at 4 °C with the anti-IA-2 N-terminal antibody (#49) or α-tubulin (1:5000 dilution). After washing, the blots were incubated for 30 min at room temperature with HRP (horseradish peroxidase) conjugated secondary antibodies. The blots were then washed and films were subsequently developed. Blots were quantitated using the NIH Image J Software.

### ACh rescues NCI-H82 cell growth

Different concentrations of ACh (0, 1, 10, 100, 300, 1, 3, and 10 mM) were added to the NCI-H82 cells, and 4 days later, a cell growth assay was performed. The optimal concentration of ACh was selected, and then added to the NCI-H82 cells treated with scramble siRNA, IA-2 siRNA, miR-342 mimic, or the miR-342 inhibitor. Four days later, a cell growth assay was performed to evaluate the rescue effect of each treatment condition.

### Statistical analysis

All experiments were performed in triplicate and the data represent the mean ± SEM. The Student’s *t* test and One-way ANOVA were used to determine statistical significance. In all cases, a P < 0.05 was considered significant.

## Results

### Downregulation of IA-2 by siRNA suppresses SCLC cells growth as well as ACh cell content and secretion

NCI-H82 cells were treated with IA-2 siRNA (Hs_PTPRN_3, 4, 5, 6) and then IA-2 mRNA and IA-2 protein levels were measured by quantitative real-time PCR (qRT-PCR) and Western Blot. Both IA-2 mRNA and protein were substantially decreased (Fig. 1a–c). Immunostaining with the IA-2 antibody confirmed the reduction of IA-2 expression after transfecting the cells with the IA-2 siRNA (Fig. 1d).

**Fig. 2:**
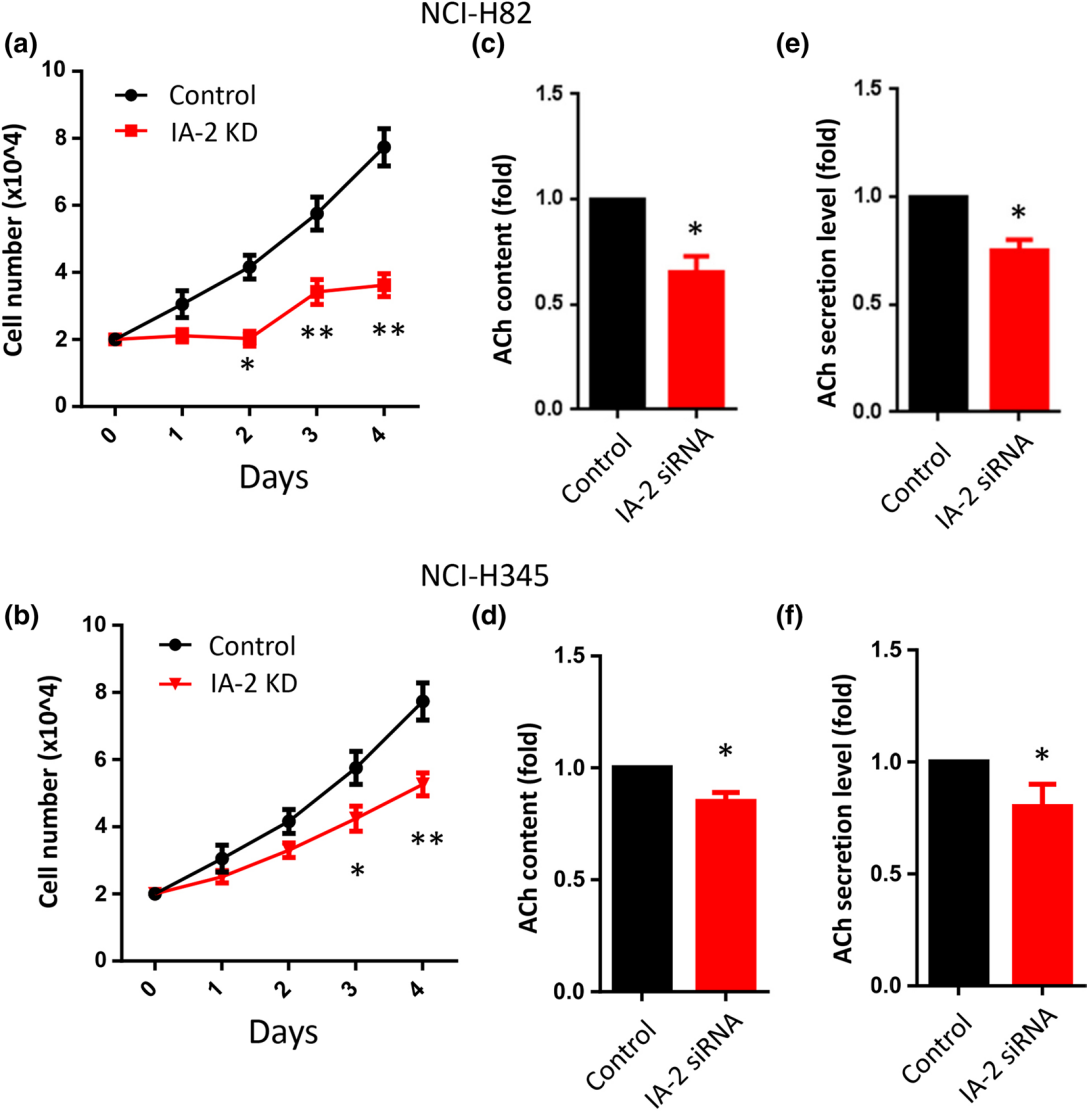
IA-2 silencing by RNAi leads to reduced SCLC cell proliferation and ACh secretion. **a**, **b** Effects of IA-2 RNAi on cell growth in both NCI-H82 and NCI-H345 cells. Cells were treated with IA-2 siRNA for 1–4 days (n = 4). Secreted ACh levels (**e**, **f**) and intracellular ACh levels (content) (**c**, **d**), were measured after four days of IA-2 siRNA (n = 3). Data (mean ± SE) were derived from three independent experiments performed in triplicate and normalized to total cell numbers. *P < 0.05, **P < 0.01

To elucidate the role of IA-2 on SCLC growth, we knocked down endogenous IA-2 in NCI-H82 and NCI-H345 cells with IA-2 siRNA. A significant reduction in cell proliferation was observed in both cell lines, with the greatest reduction occurring in the NCI-H82 cell line (60 %), compared to the NCI-H345 cell line (40 %) at 4 days post-transfection (Fig. 2a, b). Previous reports showed that ACh acts as an autocrine growth factor. To investigate whether IA-2 could regulate ACh content and secretion in SCLC cells, both were measured by ELISA after cells were transfected with IA-2 siRNA for 4 days. As seen in Fig. 2c and e, both ACh cell content and ACh secretion were decreased by ~20 and ~30 %, respectively in NCI-H82 cells, as compared to the scramble siRNA control. Similar results were observed with NCI-H345 cells (Fig. 2d, f).

### miR-342 regulates IA-2 expression in SCLC cells

**Fig. 3:**
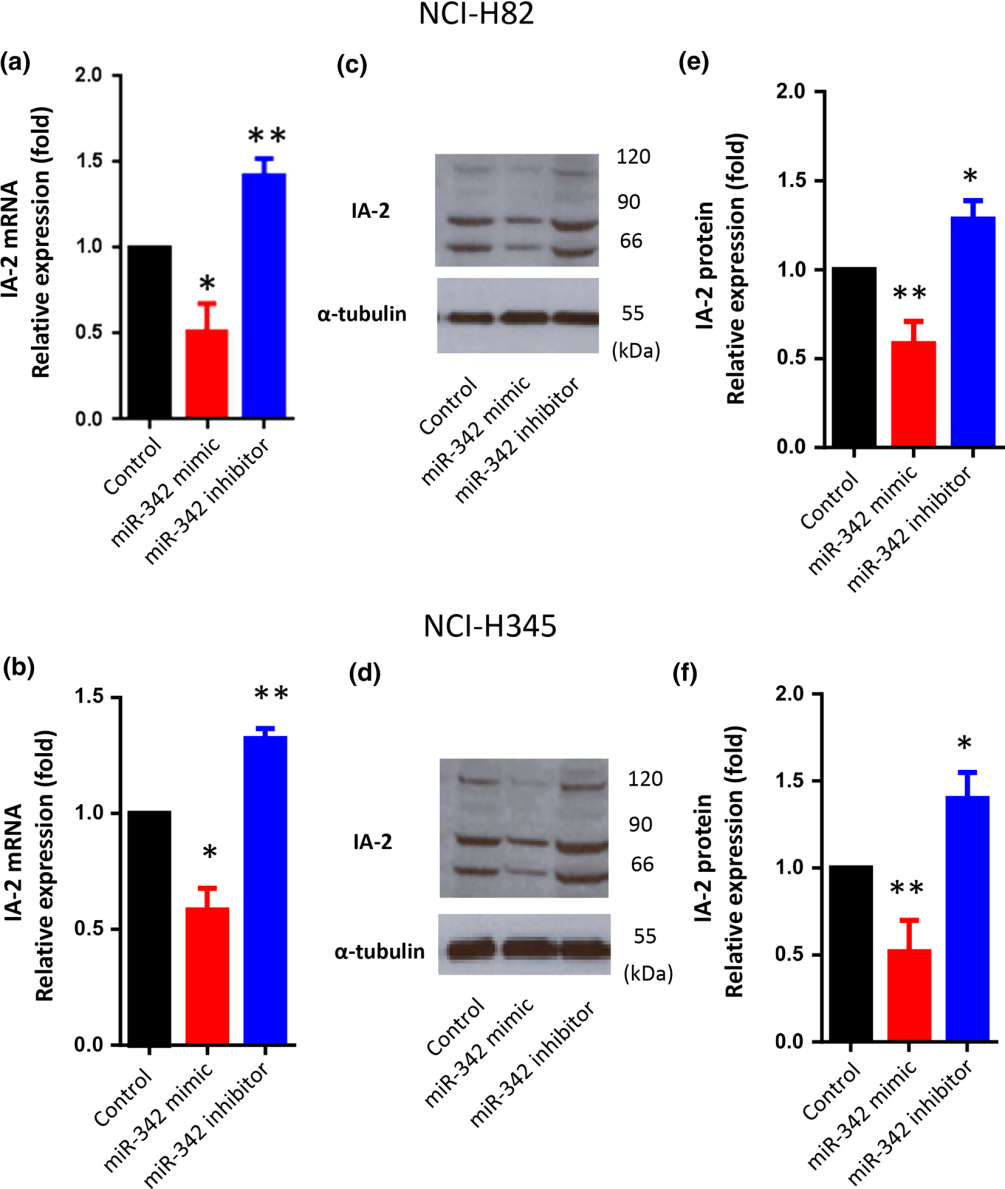
miR-342 regulates IA-2 expression level in SCLC cells. **a**, **b** Effects of miR-342 mimic and miR-342 inhibitor on IA-2 expression in NCI-H82 and NCI-H345 cells (n = 4), as quantitated by Real-Time PCR. Relative mRNA levels were normalized to GAPDH. **c**, **d** Protein levels for IA-2 in NCI-H82 and NCI-H345 cells (n = 4) were quantitated by *Western blotting* using the anti-IA-2 antibody. **e**, **f** Relative expression levels of IA-2 in *Western blots* (normalized by α-tubulin), were determined by NIH Image J. *p < 0.05; **p < 0.01

To see if the expression of IA-2 is regulated by miRNAs, NCI-H82 and NCI-H345 cells were transfected with a miR-342 mimic or miR-342 inhibitor. As seen in Fig. 3, the miR-342 mimic significantly decreased the expression of IA-2 mRNA, whereas the miR-342 inhibitor significantly enhanced the expression of IA-2 mRNA in both cell lines (Fig. 3a, b). Western blot analysis showed that miR-342 mimic decreased, whereas miR-342 inhibitor enhanced protein expression in both cell lines (Fig. 3c–f).

### MiR-342 mimic suppresses IA-2 expression, SCLC cell growth and ACh content and secretion

**Fig. 4:**
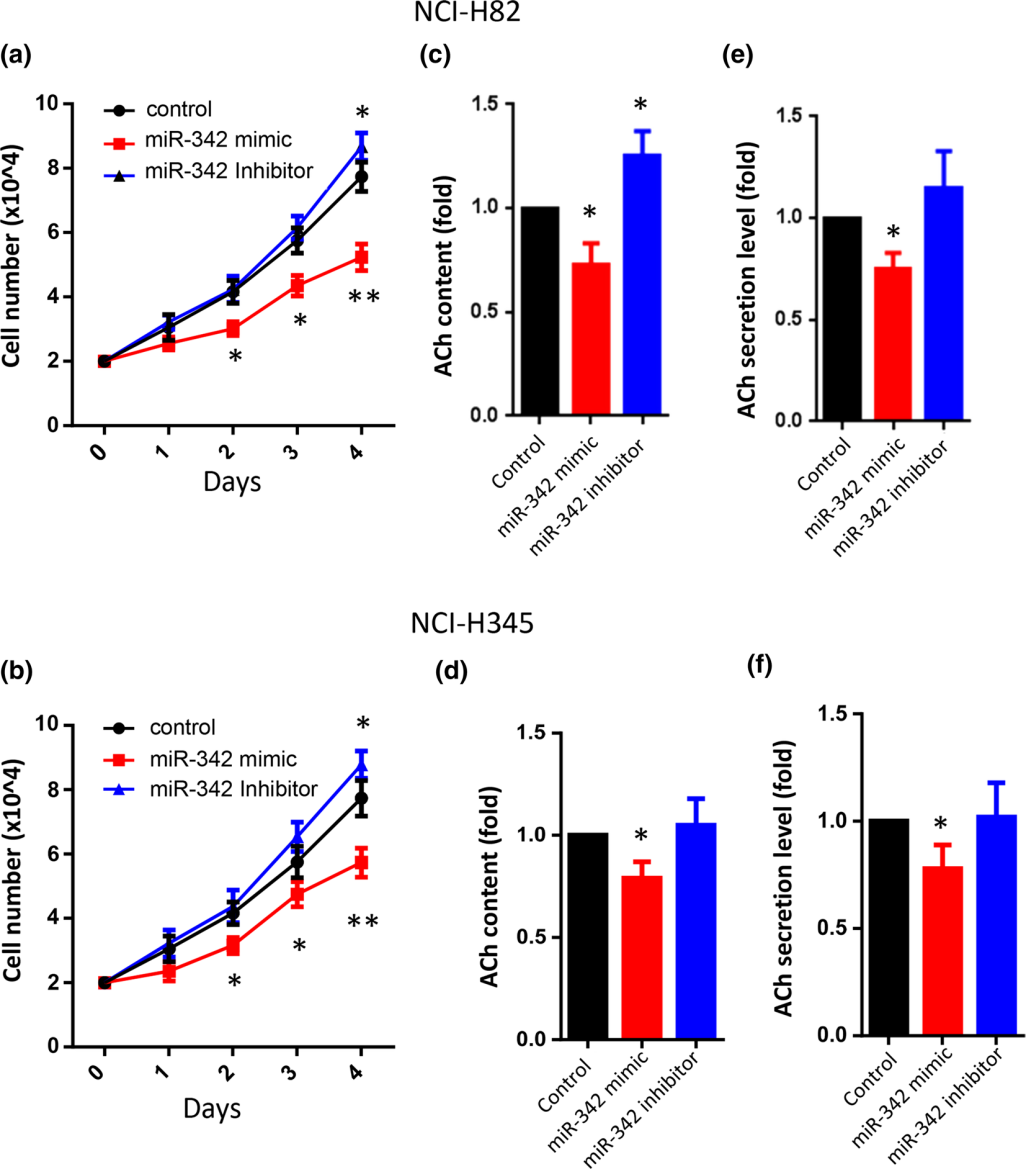
Inhibitory effect of miR-342 mimic on SCLC growth and ACh secretion. **a**, **b** Cell growth rates (based on cell count), were significantly reduced in both NCI-H82 and NCI-H345 cell lines transfected with the miR-342 mimic. A barely significant increase in cell growth was observed in the cells treated with the miR-342 inhibitor (96 h after treatment; n = 6). ACh secretion levels (**e**, **f**), and intracellular ACh content (**c**, **d**), were measured in both NCI-H82 and NCI-H345 cells after transfecting with either the miR-342 mimic or the miR-342 inhibitor 4 days post-transfection. (n = 3). Data (mean ± SE) were derived from three independent experiments (in triplicate) and normalized to total cell numbers. *P < 0.05, **P < 0.01

Given that miR-342 directly targets IA-2 as previously demonstrated in the insulin-containing MIN6 cells by “RNA immunoprecipitation PCR with anti-argonaute 2” [25], the impact of miR-342 on SCLC cell proliferation was evaluated. Growth curve analysis showed that NCI-H82 and NCI-H345 cells treated with the miR-342 mimic significantly suppressed SCLC cell proliferation by as much as 30 % (Fig. 4a, b). In contrast, the miR-342 inhibitor produced a slight but significant increase in SCLC cell proliferation at 4 days after transfection (Fig. 4a, b).

To determine the effect of miR-342 on ACh cell content and secretion, SCLC cells were transfected with a miR-342 mimic or inhibitor. Both NCI-H82 and NCI-H345 cells transfected with the miR-342 mimic showed a significant reduction in ACh cell content and secretion (Fig. 4c–f). NCI-H82 cells treated with the miR-342 inhibitor showed a slight but not significant increase in ACh secretion (Fig. 4e–f), and a barely significant increase in ACh content (Fig. 4c).

### ACh rescues the inhibitory effects IA-2 siRNA or miR-342 mimic on SCLC proliferation

**Fig. 5:**
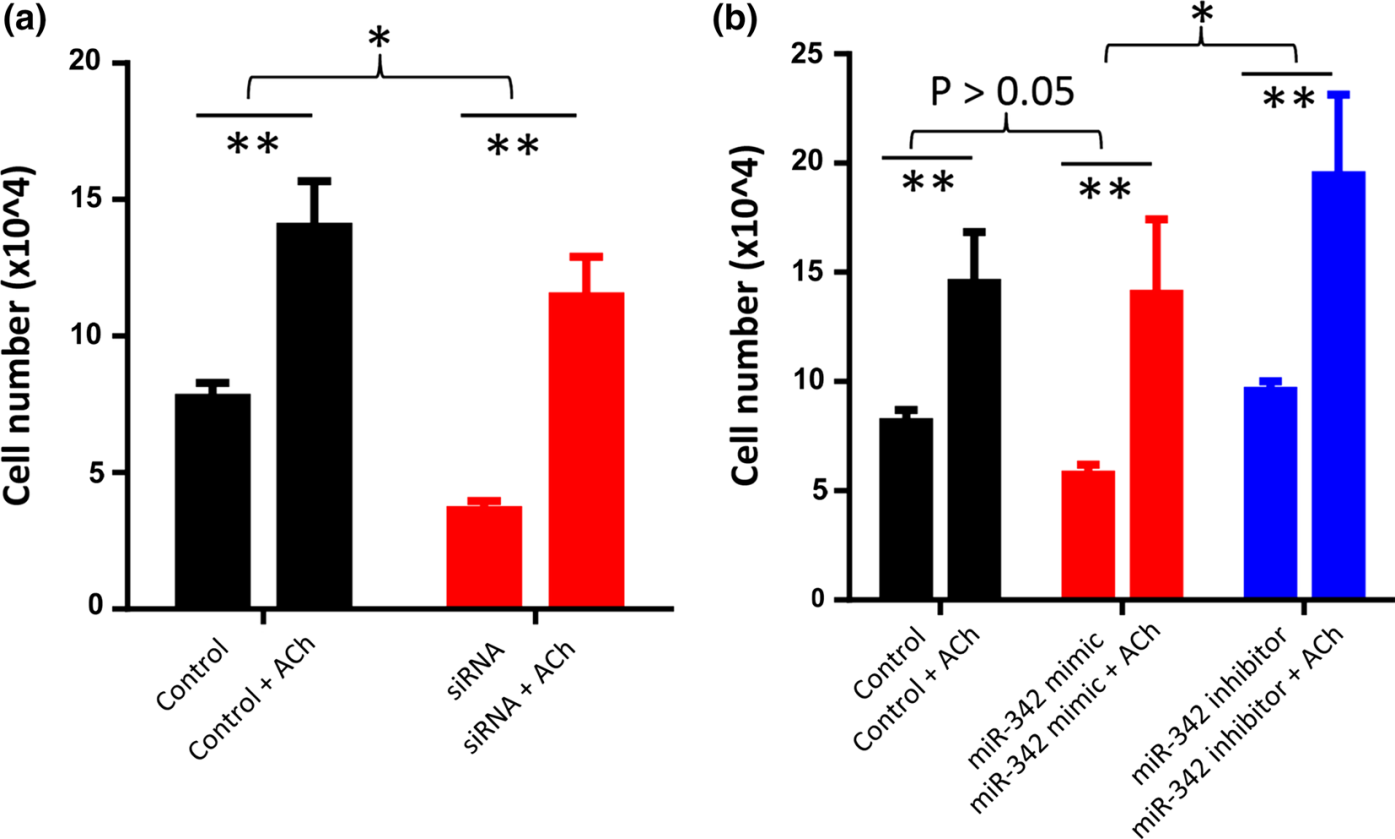
ACh rescues the inhibitory effect of IA-2 siRNA and miR-342 mimic on SCLC cell growth. **a** Treatment of SCLC cells with IA-2 siRNA inhibits cell growth. In contrast, treatment of NCI-H82 cells with ACh (1 mM), rescues cell growth. The increase of proliferation in the IA-2 siRNA and ACh treated cells is significantly higher than that incubated with ACh. **b** Similarly, the inhibitory effect of miR-342 on NCI-H82 cell growth is reduced by ACh treatment (1 mM). Similarly, the addition of ACh to cells treated with the miR-342 inhibitor produced an increase in cell proliferation with the greatest magnitude compared to the other treatment conditions. *n* = 4; *P < 0.05, **P < 0.01

To confirm that the effect of IA-2 siRNA and miR-342 on SCLC cell proliferation is mediated through the ACh autocrine growth loop, ACh was added to the culture medium of the NCI-H82 cells that were treated with either the scrambled siRNA, IA-2 siRNA, miR-342 mimic, or the miR-342 inhibitor. As seen in Fig. 5a, the addition of ACh to control cells resulted in significantly increasing the amount of cell proliferation by as much as 50 %. An even greater increase in proliferation was observed when IA-2 siRNA treated cells were incubated with ACh (1 mM, optimal concentration). Similarly, ACh produced a highly significant increase in the proliferation of cells that had been treated with miR-342 mimic (Fig. 5b). Finally, the addition of ACh to cells that had been treated with the miR-342 inhibitor produced an increase in cell proliferation with the greatest magnitude compared to the other treatment conditions (Fig. 5b). Taken together, these findings show for the first time, that the neurotransmitter ACh is effective in rescuing the inhibitory effects of siRNA and miR-342 on cell proliferation.

## Discussion

Our previous studies demonstrated that IA-2 is highly expressed in multiple human SCLC cell samples [18], but not in normal lung tissue. Although IA-2 has been well characterized in many neuroendocrine cells, its characterization and function in SCLC have so far not been determined. In this study, we found that high expression of IA-2 is associated with the growth and proliferation of SCLC cells. Conversely, we also demonstrated that the knockdown of IA-2 expression by RNAi can significantly reduce the growth rate of SCLC cells.

SCLC cells have the capacity to synthesize and secrete ACh [6, 7] and a cholinergic autocrine loop positively regulates SCLC cell growth [6, 26]. On the cytoplasmic membrane, two types of ACH receptors have been found: nAChR ligand-gated ion channels (α3, α5, α7, β2 and β4) and G protein-coupled receptors mAChR (M3 and M5) [6, 27–30]. Moreover, the knockdown of choline transporter-like protein 4 (CTL4), which mediates ACh synthesis and secretion, has been shown to significantly depress the growth of SCLC (NCI-H82) cells without affect choline uptake [31].

**Fig. 6:**
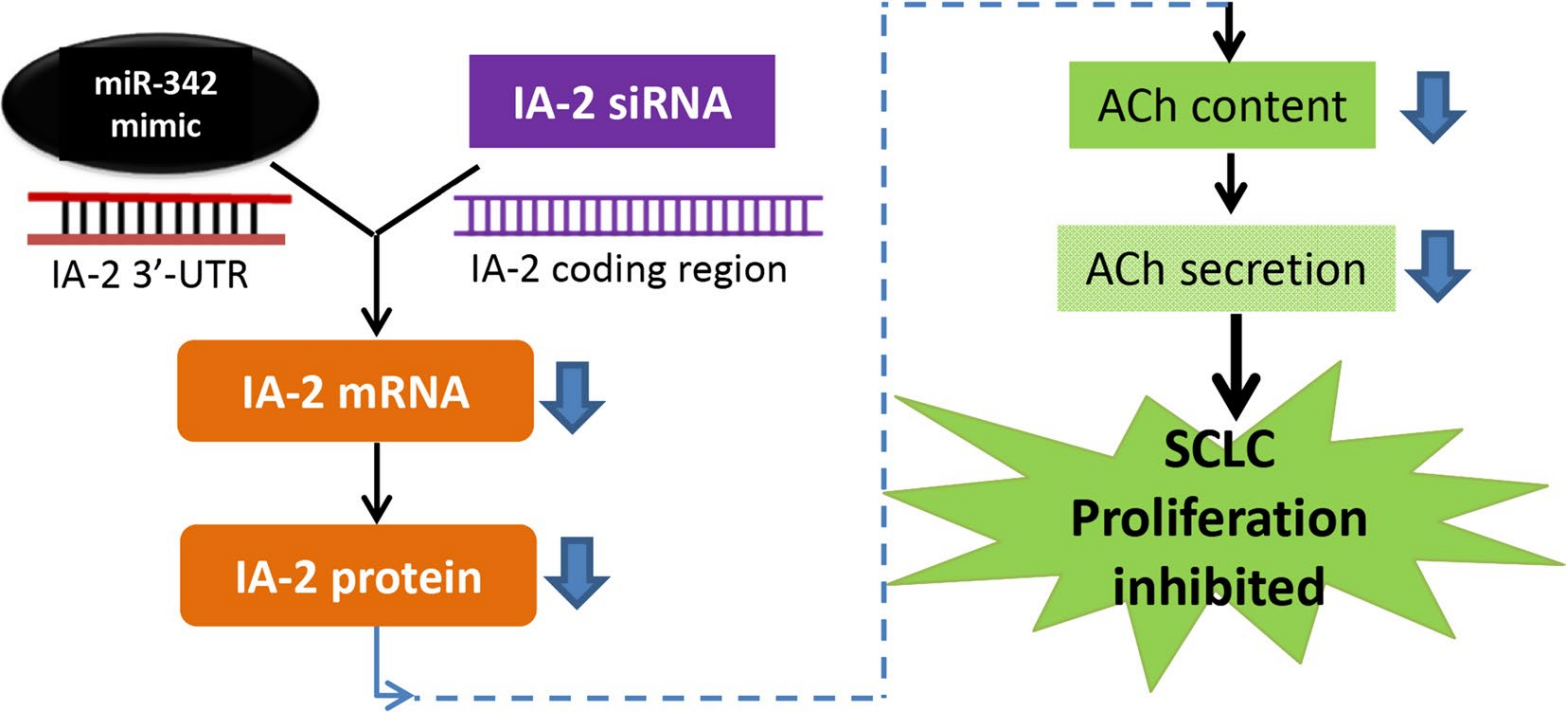
Schematic representation of the effects of IA-2 siRNA and miR-342 mimic on SCLC cell proliferation through ACh autocrine function

In the present study, we showed that silencing of IA-2 results in a substantial decrease in SCLC cell growth secondary to a decrease in the content and secretion of ACh (Fig. 6). The silencing of IA-2 has previously been shown to decrease the stability and number of DCV and, in turn, the secretion of hormones and neurotransmitters. In the present study, we also showed that overexpression of the miR-342, which targets the 3′-UTR (untranslated region) of IA-2, results in a decrease in the expression of IA-2 mRNA and, in turn, a decrease in the cellular content and secretion of ACh resulting in a decrease in the growth of SCLC cells (Fig. 6). Further support for the idea that the decrease in ACh is responsible for the decrease in SCLC cell growth was obtained by restoring cell growth to nearly normal levels by treatment with ACh.

SCLC is an aggressive malignancy with a distinct natural history and poor prognosis, leading to the death of approximately 16,000 patients per year in United States. For decades, cytotoxic chemotherapy has remained the backbone of treatment since SCLC is a chemo-sensitive tumor. Nonetheless, high response rates are not usually translated into cure. In spite of recent advances in elucidating aberrant molecular pathways and new therapeutic targets, no significant improvement in patients’ survival has been demonstrated [32].

## Conclusion

Taken together, the demonstration that both miR-342 and its target gene IA-2 are involved in the growth of SCLC cells, through their effect on the secretion of acetylcholine, represents a novel approach that may be therapeutically useful in the treatment of SCLC tumors.

## Abbreviations

ACh: acetylcholine
ACTH: adrenocorticotropic hormone
CCK-8: cell counting kit-8
DAPI: 4′,6-diamidino-2-phenylindole, dihydrochloride
DCV: dene-core vesicle
ELISA: enzyme-linked immunosorbent assay
FSH: follicular stimulating hormone
GAPDH: glyceraldehyde-3-phosphate dehydrogenase
NIH: National Institutes of Health
HRP: horseradish peroxidase
IA-2: islet cell antigen-2
ICA512: islet cell antigen 512
LH: luteinizing hormone
NIDCR: National Institute of Dental and Craniofacial Research
NE: norepinephrine
NSCLC: non-small cell lung cancer
Phogrin: phosphatase homologue in granules of insulinoma
POMC: pro-opiomelanocortin
PTPRN: protein tyrosine phosphatase, receptor type N
PVDF: polyvinylidene fluoride
SCLC: small cell lung cancers
UTR: untranslated region
